# Characteristics and Prognosis of COVID-19 in Patients with COPD

**DOI:** 10.3390/jcm9103259

**Published:** 2020-10-12

**Authors:** Desirée Graziani, Joan B Soriano, Carlos Del Rio-Bermudez, Diego Morena, Teresa Díaz, María Castillo, Miguel Alonso, Julio Ancochea, Sara Lumbreras, José Luis Izquierdo

**Affiliations:** 1 Respiratory Medicine, University Hospital of Guadalajara, 19002 Guadalajara, Spain; desygraziani@gmail.com (D.G.); diegomorenavalles6@gmail.com (D.M.); Mariacastillogarcia37@gmail.com (M.C.); alonsomiguel23@gmail.com (M.A.); 2 Respiratory Medicine, Hospital Universitario de La Princesa, 28006 Madrid, Spain; jbsoriano2@gmail.com (J.B.S.); juli119@gmail.com (J.A.); 3 Universidad Autónoma de Madrid, 28049 Madrid, Spain; 4 Respiratory Diseases Networking Biomedical Research Centre (CIBERES), Institute of Health Carlos III (ISCIII), 28006 Madrid, Spain; 5 SAVANA Medica, 28013 Madrid, Spain; cdelrio@savanamed.com (C.D.R.-B.); tdiaz@savanamed.com (T.D.); 6 Universidad Pontificia Comillas, 28015 Madrid, Spain; slumbreras@savanamed.com; 7 Department of Medicine and Medical Specialties, University of Alcalá, 28801 Madrid, Spain

**Keywords:** COPD, COVID-19, prevalence, prognosis

## Abstract

Patients with Chronic Obstructive Pulmonary Disease (COPD) have a higher prevalence of coronary ischemia and other factors that put them at risk for COVID-19-related complications. We aimed to explore the impact of COVID-19 in a large population-based sample of patients with COPD in Castilla-La Mancha, Spain. We analyzed clinical data in electronic health records from 1 January to 10 May 2020 by using Natural Language Processing through the SAVANA *Manager*^®^ clinical platform. Out of 31,633 COPD patients, 793 had a diagnosis of COVID-19. The proportion of patients with COVID-19 in the COPD population (2.51%; 95% CI 2.33–2.68) was significantly higher than in the general population aged >40 years (1.16%; 95% CI 1.14–1.18); *p* < 0.001. Compared with COPD-free individuals, COPD patients with COVID-19 showed significantly poorer disease prognosis, as evaluated by hospitalizations (31.1% vs. 39.8%: OR 1.57; 95% CI 1.14–1.18) and mortality (3.4% vs. 9.3%: OR 2.93; 95% CI 2.27–3.79). Patients with COPD and COVID-19 were significantly older (75 vs. 66 years), predominantly male (83% vs. 17%), smoked more frequently, and had more comorbidities than their non-COPD counterparts. Pneumonia was the most common diagnosis among COPD patients hospitalized due to COVID-19 (59%); 19% of patients showed pulmonary infiltrates suggestive of pneumonia and heart failure. Mortality in COPD patients with COVID-19 was associated with older age and prevalence of heart failure (*p* < 0.05). COPD patients with COVID-19 showed higher rates of hospitalization and mortality, mainly associated with pneumonia. This clinical profile is different from exacerbations caused by other respiratory viruses in the winter season.

## 1. Introduction

Chronic Obstructive Pulmonary Disease (COPD) is one of the most prevalent chronic diseases, one of the main diagnoses in hospital admissions (especially during winter), and the fourth leading cause of death worldwide. Importantly, one of the main factors underlying the negative impact of the disease in patients and health systems is COPD exacerbation [1]. In turn, these exacerbations are primarily caused by respiratory viral infections (especially during epidemic periods), which have a direct effect on the symptomatology and favor bacterial superinfections [2,3]. COPD exacerbations worsen the prognosis of the disease by increasing mortality when associated with hospitalizations [4].

Caused by coronavirus-2 (SARS-Cov-2), the clinical manifestation of COVID-19 varies from mild to very severe symptoms and can lead to death in some patients [5,6]. Since the onset of the COVID-19 pandemic, the severity of the disease has been associated with pre-existing comorbidities, namely cardiovascular diseases, diabetes mellitus, and hypertension. In contrast with the reported burden of influenza epidemics in COPD patients, the impact of COVID-19 in these patients seems to have been less evident; however, COPD patients usually present a fragile situation and a lower respiratory functional reserve [7,8].

COVID-19 severity and mortality have also been associated with patient’s age. Although the virus can infect individuals of all ages, the majority of severe cases to date have been described in people older than 55 years and with significant comorbidities [9,10]. The prevalence of COPD also increases markedly with age, with most diagnoses occurring in patients aged 40 and older. Indeed, patients over 40 years old frequently present with more than one chronic disease, especially in the endocrine-metabolic and cardiovascular spheres. Several observational and case-control studies have confirmed a higher prevalence of cardiovascular diseases in COPD patients than in the general population, possibly due to the coexistence of common risk factors or an associated pathogenic mechanism [11]. Although there are large discrepancies in the studies that have evaluated the relationship between COPD and cardiovascular disease, COPD patients undoubtedly have a higher prevalence of coronary ischemia and other risk factors that may worsen the prognosis of COVID-19 [12]. Based on the above, it is crucial to characterize the evolution of SARS-Cov-2 infection in COPD patients and identify the impact of COPD and associated comorbidities in the patient’s evolutionary course.

The combination of real-world data (RWD) with big data analytics and artificial intelligence has the potential to increase our understanding of COVID-19 in a timely manner. Using such methods, this study aims to reuse the clinical information contained in the electronic health records (EHRs) of the population with COPD and COVID-19 to (a) describe the clinical characteristics of patients with COPD and COVID-19 and (b) to assess the influence of COPD and related comorbidities and treatments in the prognosis of COVID-19.

## 2. Methods

This was a multicenter, non-interventional, retrospective study using free text data captured in the EHRs of patients diagnosed with COPD and COVID-19. The study period was 1 January–10 May 2020. We followed the Strengthening the Reporting of Observational Studies in Epidemiology (STROBE) guidelines for reporting observational studies [13].

Clinical data from a total of 1,164,283 patients with available EHRs throughout the community of Castilla La-Mancha (Spain) were collected from all available departments, including inpatient, outpatient, emergency room, and primary care.

Natural Language Processing (NLP) and artificial intelligence (AI) techniques were used to extract and analyze the information in EHRs. The software used (SAVANA *Manager*^®^) is a powerful multilingual, free-text analysis engine capable of interpreting the content in clinical records, regardless of the system in which they operate. The software can capture numerical values and physician’s notes and translate them into usable variables, thus allowing the reuse of information included in large-scale collections of clinical records; therefore, the processed free-text information captured in EHRs is treated as Big Data. The methodology used to generate the study database has four distinct phases for data extraction and aggregation, namely (a) Acquisition: the acquisition of data is the responsibility of the healthcare center, in close collaboration with the staff of Savana Information Technology. Following the General Data Protection Regulation (GDPR) of the European Union, data are extracted, anonymized, and transferred to Savana; (b) Integration: In this phase, data are integrated into the database; (c) NLP processing: Using the EHRead^®^ technology developed by Savana, NLP techniques are implemented to analyze and extract the unstructured, free-text information written in millions of EHRs. The output of NLP processing is a synthetic patient database, as the software creates a patient database from scratch. This ensures that this information is protected and makes traceability to individual patients impossible; (d) Validation: this process consists of a medical validation carried out by doctors and researchers. The terminology used by Savana is based on multiple sources, such as SNOMED CT [14]. This terminology includes codes, concepts, synonyms, and definitions used in clinical documentation. It also includes symptoms, diagnoses, body structures, and substances. Due to the novel methodological approach of this study, we complemented our clinical findings with an evaluation of Savana’s performance. This evaluation aims to verify the precision of the system by identifying records that contain mentions of COPD, COVID-19, and its related variables. The results of the annotations were used to generate the gold standard and to calculate Savana’s performance. The performance of the system is calculated in terms of the standard metrics for precision (P), recall (R), and its harmonic mean F-score [15]:

Precision = tp/(tp + fp). This parameter gives us an indicator of the precision of the information that the system retrieves.

Recall = tp/(tp + fn). This parameter gives us an indicator of the amount of information that the system retrieves.

F-Score = 2 × Precision × Recall/(Precision + Recall). This parameter provides us with a general performance indicator for information retrieval.

In all cases, tp is the number of true positives (i.e., records retrieved successfully), fn is the number of false negatives (i.e., records incorrectly not retrieved), and fp is the number of false positives (i.e., records recovered incorrectly).

The search terms for COPD and COVID-19 have been previously described [16,17]. For the linguistic evaluation of the variable “COPD”, we obtained Precision, Recall, and F-Scores of 0.926, 0.895, and 0.912, respectively; these metrics indicate that patients with COPD were properly identified within the target population. *EHRead* identified COVID-19 with a Precision of 0.99, a Recall of 0.75, and an F-Score of 0.93; again, these results indicate that within our population with COPD, COVID-19 cases were accurately identified.

For all statistical analyses, SPSS software (v 25.0) was used. Categorical variables are reported as absolute frequencies and percentages, while continuous variables are presented using mean and standard deviation. For the assessment of statistical significance of numerical variables, we used *t*-tests for independent samples or ANOVAs. To measure the relative distribution of patients assigned to different categories of qualitative variables, we used Chi^2^ tests. In all cases, a *p* value for statistical significance was set at 0.05.

The study was compliant with legal and regulatory requirements and the research practices described in the ICH Guide to Good Clinical Practice, The Declaration of Helsinki in its latest edition, good pharmacoepidemiology practices, and local regulations. Since this is a retrospective and observational study using anonymous patient data, informed consent does not apply to the present study. All actions were taken following the code of good data protection practices for Big Data projects of the European Data Protection Authority and the European GDPR. The study has been approved by the ethics and research committee of the University Hospital of Guadalajara (Spain).

## 3. Results

**Figure 1:**
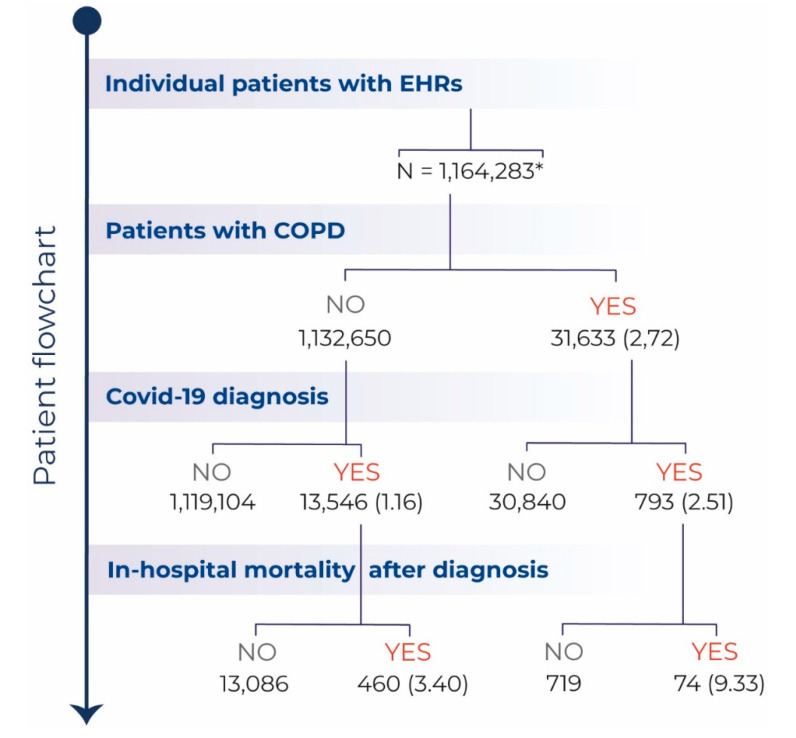
Patient Flowchart. Flowchart depicting the total number (%) of patients with available electronic health records (EHRs) during the study period (1 January 2019–10 May 2020), the number of patients with COPD seen during this period, the number of patients diagnosed with COVID-19, and of those, the number of deaths after diagnosis. All percent values are computed in relation to the level immediately above. * Patients aged > 40 years.

A total of 31,633 patients with a diagnosis of COPD were attended by the health system of Castilla La-Mancha (Spain) between 1 January 2019 and 10 May 2020. Among these, 793 patients were diagnosed with COVID-19. The patient flowchart is depicted in Figure 1.

The percentage of patients diagnosed with COVID-19 in the COPD population (2.51%; 95% CI 2.33–2.68) was significantly higher than in the general population older than 40 years, (1.16%; 95% CI 1.14–1.18); *p* < 0.001. COVID-19 diagnosis was confirmed by PCR in 335 (42%) of patients; in the remaining cases, diagnosis was based on rapid serological tests or clinical, radiological, and/or analytical evaluation, considering the reduced availability of PCR testing in the study area between March and May 2020. The demographic and clinical characteristics of COVID-19 patients with and without COPD are shown in Table 1. Compared with COPD-free patients with COVID-19 older than 40 years, patients with both COVID-19 and COPD were older ((mean age ± SD) 75 ± 12 years vs. 66 ± 15 years (*p* < 0.001)) and predominantly male. Furthermore, these patients showed a higher prevalence of comorbidities and a worse prognosis, as evaluated by hospitalizations (31.1 % vs. 39.8%: OR 1.57; 95% CI 1.14–1.18) and mortality rate (3.4% vs. 9.3%: OR 2.93; 95% CI 2.27–3.79) (Table 1).

In Castilla-La Mancha, the COVID-19 pandemic began in March 2020. At that time, there were hardly any other viral infections, including influenza. To assess the burden of COVID-19 in patients with COPD, we compared the clinical characteristics and outcomes of patients with COPD and COVID-19 during the study period with existing data from COPD patients during the last two winter seasons. Although patients’ characteristics (including comorbidities) were similar in the two periods, COVID-19 was associated with poorer prognosis in terms of hospitalization and mortality (Table 2).

The main diagnosis of COPD patients with COVID-19 requiring hospital admission was pneumonia (59% of patients); 19% of hospitalized patients had pulmonary infiltrates, in turn suggestive of pneumonia and heart failure. In patients who died, the distribution of patients with pneumonia and heart failure were 72% and 28%, respectively, similar to non-COPD patients with COVID-19 who died (71% had pneumonia and 24 pulmonary infiltrates with different diagnosis, mainly heart failure or acute respiratory distress syndrome). In the COPD population with COVID-19, those who died were older (77 ± 11 years vs. 74 ± 11 years; *p* = 0.03) and had a higher incidence of heart failure than those who did not die from the disease; no other prognostic factor was identified (Table 3).

Regarding pharmacological treatment, most patients were under treatment with bronchodilators, namely beta 2 agonists and anticholinergics (Table 4); a significantly greater use of both drugs was observed in those patients who died. On the other hand, the elevated rates of inhaled steroids use in both patient groups forbid further assessment of differences regarding the use of these agents and mortality. Finally, we did not observe any differences in COVID-19-related mortality rates based on use of cardiovascular drugs.

Finally, in Table 5, our finding observed in the crude analysis of an association with death in COVID-19 patients with COPD is further confirmed. The increased mortality risk of COVID-19 patients with COPD versus those without COPD is sustained in several multivariate analyses adjusted by covariates, and very consistently, sequentially with OR and 95% CI of 1.70 (1.29–2.23) adjusted by sex and age (Model 1); of 1.52 (1.15–2.00) when we also added the two most relevant comorbidities, that is heart failure and high blood pressure (Model 2); and of 1.42 (1.07–1.88) when we added all single comorbidities in a full model (Model 3).

## 4. Discussion

Since the WHO declared the COVID-19 outbreak a global pandemic, clinicians have aimed at determining the impact of the disease on patients with chronic diseases, especially of pulmonary and cardiovascular nature. Although the frequency and severity of COVID-19 has been associated with pre-existing comorbidities such as heart disease, arterial hypertension, and diabetes. Surprisingly and “against all prognosis”, however, healthcare data show that the incidence of COVID-19 in COPD patients has been relatively low [18]. This trend was already observed from the onset of the pandemic; a study that evaluated 1590 hospitalized patients with COVID-19 in China revealed a low incidence in patients with COPD, with a total of 24 cases [19]. However, COPD was linked with a higher risk for poor disease outcome (composite endpoint including admission to an intensive care unit, invasive ventilation, or death), reflected by a hazard ratio (HR) of 2681 (95% CI 1424–5.0480), after adjusting for age and smoking. In this study, the comorbidity of COPD as a risk factor was exceeded only by malignancy (HR 3.50, 95% CI 1.60 to 7.64) [19]. Subsequently, a systematic review and meta-analysis showed that, although the prevalence of COPD in COVID-19 cases was low, SARS-CoV-2 infection was associated with high rates of severity and mortality in patients with COPD [20].

In our study, we have confirmed that the impact of COVID-19 in COPD patients has been relatively limited. Plausible underlying reasons for this include remission of the seasonal flu period, an absence of exposure to environmental factors due to isolation, the significant drop in contamination, and better control of the disease by complying with the treatments conscientiously “out of fear”. Our results, however, indicate that patients with COPD are at a higher risk of SARS-CoV-2 infection doubling the infection rates observed in the general population over 40 years of age. This increased risk has also been described in a concise meta-analysis showing that COPD is associated with a significant, five-fold increased risk of severe COVID-19 infection; of note, this analysis is focused on the Chinese population and there was substantial variability among the included studies [21].

Although a greater risk associated with COVID-19 in COPD patients seems clear, it is difficult to accurately determine the extent to which COPD itself or associated comorbidities affect the higher prevalence of COVID-19 and its prognosis. In our study, COPD patients were older and had more comorbidities. These factors could have been critical in the reported higher rates of admissions and mortality. Although it is not possible to accurately assess the impact of comorbidities in COPD [12] in a cross-sectional study, there is no doubt that COPD patients are at higher risk for COVID-19, showing a higher incidence of the disease and worse prognosis (as determined by higher hospitalization and mortality rates). Our multivariate analyses presented in Table 5 adds strength to our finding of an increased risk of death, ranging from 72% to 42%, in COVID-19 patients with COPD versus those without COPD. Please note that in the full model 3, some individual comorbidities were associated with nominal increases, but not statistically significant increased risks, such as stroke, ischemic heart disease, ischemic heart disease, and sleep apnoea, as well as smoking with an odds ratio (OR) of 1.31 95% confidence interval (CI) (0.99–1.71), likely due to mathematical collinearity of these comorbidities in multimorbid patients.

Given the scarcity of PCR tests for SARS-Cov-2 at the onset of the COVID-19 pandemic, regional protocols established the performance of multiple PCR tests for respiratory viruses in all patients who were hospitalized for respiratory symptoms. The results of these tests allowed us to confirm the near absence of other viral infections, including influenza, during the COVID-19 pandemic. This allowed us to compare the differential impact of COVID-19 in COPD patients by comparing data during this period with data from the last two winter seasons. Although both populations showed similar age and comorbidities, COVID-19 caused a higher rate of hospitalizations and in-hospital mortality. Most patients admitted for COVID-19 presented pulmonary infiltrates compatible with SARS-CoV-2 pneumonia and, in some cases, with associated heart failure; this finding markedly differed from patients with COPD exacerbation due to other viral causes. These data indicate that patients who were admitted into a hospital or died from COVID-19 have different clinical profiles compared to those with winter viral exacerbations. Thus, these differences must be taken into account so we can adapt to a scenario where both clinical profiles can coexist. As Faust comments in a recent article [22], many hospitals in areas hard-hit by COVID-19 (as is the case in our study population) have had an unprecedented overload and demand for hospital resources during the crisis that have never been seen before, even during the worst flu season. Although the interaction that may exist between SARS-CoV-2 and other viruses is unknown, the scenario can be further complicated by their simultaneous presentation.

The treatment of patients with chronic diseases have been another highly discussed topic with regards to the newly identified pandemic. Faced with the initial alarm regarding the deleterious potential of certain drugs such as ACE inhibitors or ARBs, our data do not confirm a negative impact of these drugs in patients with COPD and related comorbidities that justify their use. These data are consistent with other series in the general population [23,24]. In patients with COPD and asthma, although there is still very little scientific evidence, the treatment with inhaled glucocorticoids (IGC) could have a “protective effect” against COVID-19 since they may decrease the expression of the ACE2 receptor genes and the TMPRSS2 transmembrane protease genes, both key for the virus to enter cells and make copies of itself [25]. Previous clinical data in patients with asthma support this hypothesis in our population [17]. However, since most COPD patients were under treatment with IGCs, it was not possible to evaluate the specific effect of IGCs in our population. Since the prescription of beta 2 agonists and anticholinergics is guided by symptoms, the observed greater use in patients who died may simply be related to increased severity of the disease.

Perhaps one of the most controversial topics around COVID-19 is the association between smoking and the manifestation of the disease. Although a protective effect of nicotine was initially suggested, several meta-analyses have confirmed that smoking increases the risk of severe COVID-19 and mortality; these results are not consistent, however [20,26]. As is the case with the present study (where we were not able to determine with precision whether smokers were still active tobacco users or the intensity of exposure) the exact duration of smoking was not reported in most studies included in these meta-analyses. Both COPD and tobacco smoke can up-regulate ACE-2 expression in lower airways, which in part may explain the increased risk of severe COVID-19 in this population [27].

The results presented here must be interpreted in light of some strengths and limitations. Data were extracted from the public health system of Castilla-La Mancha, with a population of 2,030,807 inhabitants. Specifically, we analyzed data from the SESCAM health system, which operates the SAVANA *Manager*^®^ clinical platform with available data since 2011. The information obtained from 1,164,283 patients with available EHRs available during the study period is verifiable and includes the clinical management of all patients without any type of bias. This differs from other databases, where limited reliability has generated controversy [23]. Importantly, since we collected information from the entire population, reproducibility of the results does not apply. 

On the other hand, the results reported for some variables rely on the quality of the data captured in the clinical reports, which in many cases may not include all the clinical information for a given patient. Since this is not a study based on a strict registry of variables, the information that was not adequately documented was excluded from further analyses [28,29].

In this study, we included COVID-19 cases both confirmed with PCR or serological tests and those exclusively diagnosed based on clinical criteria (i.e., symptoms, imaging, and laboratory results). However, it should be noted that PCR and other rapid laboratory tests for the detection of SARS-CoV-2 were not used routinely in Spain during the onset of the pandemic. Furthermore, this decision is supported by reports questioning the clinical validity and high sensitivity of symptom and image-based identification of patients with COVID-19, especially in the early stages of the disease [30,31,32]. Finally, diagnoses for viral infections analyzed in previous winter seasons were mostly based on clinical evaluations, without specifications regarding the type of virus in most cases. The data, which spans two annual periods, are nevertheless representative of COPD exacerbations due to viral processes during the winter season.

In conclusion, the results of this study confirm a higher incidence of COVID-19 in COPD patients and higher rates of hospital admissions and mortality, mainly associated with pneumonia. This clinical profile is different from that observed during winter exacerbations caused by other respiratory viruses. Overall, our data support the implementation of specific plans in this population with close monitoring of COPD patients in sceneries at risk for COVID-19.

## Figures and Tables

**Table 1:** Characteristics and clinical outcomes of the study population.

Characteristics of the Study Population	COVID-19 > 40 Years (1 January–10 May 2020)	COPD-COVID-19 (1 January–10 May 2020)	OR (95% CI) *p*-Value
N	13.546	793	
Age			
Years (mean ± SD)	66 ± 15	75 ± 12	<0.001
Sex			
Female (%)	53	17	0.18 (0.15–0.22)
Comorbidities			
Ischemic heart disease (%)	8.8	22.6	3.02 (2.53–3.60)
Heart failure (%)	13.8	33.9	3.21 (2.75–3.75
Cardiac arrhythmia (%)	8.0	18.5	2.61 (2.16–3.15)
Diabetes mellitus (%)	26.1	40.6	1.93 (1.67–2.24)
Arterial Hypertension (%)	54.2	76.4	2.74 (2.32–3.24)
Dyslipidemia (%)	15.4	20.2	1.39 (1.16–1.66)
Pulmonary embolism (%)	2.2	5.2	2.46 (1.76–3.44)
Stroke (%)	8.3	13.1	1.46 (1.18–1.81)
Smoking (%)	13	33.9	3.43 (2.93–4.00)
Hospitalization (%)	31.1	39.8	1.57 (1.35–1.82)
In-hospital mortality (%)	3.4	9.3	2.93 (2.27–3.79)

**Table 2:** Differences in characteristics and clinical outcomes between COVID-19 and influenza in patients with COPD.

Characteristics of the Study Population	COPD-COVID-19 (1 January–10 May 2020)	COPD-Influenza 2019–2020 (1 December 2018–30 April 2019)	OR (95% CI) *p*-Value *	COPD-Influenza 2018–2019 (1 December 2017–30 April 2018)	OR (95% CI) *p*-Value **
N	793	826		1066	
AgeYears (mean ± SD)	75 ± 12	72 ± 13		73 ± 13	<0.001
Sex					
Female (%)	17	19	0.87 (0.68–1.13)	17	1.00 (0.79–1.28)
Comorbidities					
Ischemic Heart disease (%)	22.6	20.8	1.11 (0.88–1.40)	23.0	0.98 (0.79–1.22)
Heart failure (%)	33.9	30.5	1.17 (0.95–1.44)	37.1	0.87 (0.72–1.05)
Cardiac arrhythmia (%)	18.5	20.5	0.89 (0.69–1.13)	22.1	0.74 (0.59–0.93)
Diabetes mellitus (%)	40.6	38	1.12 (0.91–1.36)	43.1	0.90 (0.75–1.09)
Arterial Hypertension (%)	76.4	74.5	1.11 (0.89–1.40)	77.9	0.92 (0.74–1.15)
Dyslipidemia (%)	20.2	22.9	0.85 (0.67–1.08)	21.4	0.93 (0.93–1.17)
Pulmonary embolism (%)	5.2	5.0	1.04 (0.67–1.63)	6.4	0.80 (0.54–1.19)
Stroke (%)	13.1	12.0	1.11 (0.83–1.49)	12.8	1.03 (0.79–1.36)
Smoking (%)	33.9	43.8	0.66 (0.54–0.81)	39.3	0.79 (0.65–0.96)
Hospitalization (%)	39.8	5.9	10.51 (7.61–14.5)	6.8	9.01 (6.83–11.89)
In-hospital mortality (%)	9.3	2.2	4.62 (2.73–7.81)	5.2	1.89 (1.32–2.18)

* COPD-COVID-19 vs. COPD Influenza 2019-2020. ** COPD-COVID-19 vs. COPD Influenza 2018–2019.

**Table 3:** Impact of comorbidities on the mortality of patients with COPD.

Characteristics of the Study Population	COPD-COVID-19 Survivors (1 January–10 May 2020)	COPD-COVID-19 Deceased (1 January–10 May 2020)	OR (95% CI) *p*-Value
N	719	74	
Age			
Years (mean ± SD)	74 ± 11	77 ± 11	0.03
Sex			
Female (%)	18	16	1.58 (0.37–3.25)
Comorbidities			
Ischemic Heart disease (%)	24	16	1.59 (0.84–3.02)
Heart failure (%)	33	45	0.61 (0.37–0.99)
Arrythmia (%)	18	22	0.81 (0.45–1.45)
Diabetes mellitus (%)	41	41	1.00 (0.62–1.63)
Arterial Hypertension (%)	76	77	0.96 (0.55–1.70)
Dyslipidemia (%)	20	23	0.83 (0.47–1.47)
Pulmonary embolism (%)	5	5	0.95 (0.39–1.46)
Stroke (%)	11	16	0.76 (0.58–1.71)
Sleep Apnea (%)	11	11	1.06 (0.49–2.29)
Smoking (%)	34	28	1.33 (0.78–2.25)

**Table 4:** Impact of treatments on the mortality of patients with COPD.

	COPD-COVID19 Survivors (1 January–10 May 2020)	COPD-COVID-19 Deceased (1 January–10 May 2020)	OR (95% CI)
N	719	74	
Treatment			
Inhaled steroids (%)	96	95	0.68 (0.23–2.01)
Anticholinergics (%)	89	99	8.88 (1.22–64.81)
Beta-2 agonists (%)	85	95	3.06 (1.09–8.56)
Statins (%)	48	42	0.77 (0.48–1.26)
IECAS (%)	33	23	0.60 (0.34–1.05)
ARA2 (%)	37	41	1.15 (0.71–1.88)
Antiaggregant (%)	79	74	0.77 (0.44–1.34)
Beta-blockers (%)	27	27	1.00 (0.59–1.72)

**Table 5:** Risk of death (OR and 95% CI) in COVID-19 patients with COPD versus those without COPD adjusted by covariates in several models of logistic regression multivariate analyses.

Model 1	Model 2	Model 2			
COPD	1.70 (1.29–2.23)	COPD	1.52 (1.15–2.00)	COPD	1.42 (1.07–1.88)
Sex	1.86 (1.53–2.26)	Sex	1.87 (1.54–2.28)	Sex	1.82 (1.49–2.22)
Age	1.06 (1.05–1.07)	Age	1.05 (1.04–1.06)	Age	1.05 (1.04–1.06)
		HF	1.65 (1.33–2.03)	Heart failure	1.46 (1.17–1.81)
		HBP	1.59 (1.24–2.04)	HBP	1.43 (1.10–1.84)
				Stroke	1.02 (0.78–1.32)
				Arrythmia	1.30 (1.01–1.71)
				IHD	1.05 (0.81–1.36)
				Diabetes	1.23 (1.01–1.50)
				HL	1.03 (0.81–1.31)
				Sleep apnoea	1.27 (0.85–1.90)
				PTE	1.72 (1.08–2.75)
				Smoking	1.31 (0.99–1.71)

Reference values are female sex and no comorbidity. Age is a continuous variable increasing in years; HF: Heart failure; HBP: High blood pressure; IHD: Ischemic heart disease; HL: Hyperlipidemia; PTE: Pulmonary thromboembolism.
